# The state of play: our experience integrating psychosocial support into retinoblastoma care

**DOI:** 10.1038/s41433-025-03913-z

**Published:** 2025-07-28

**Authors:** Cindy Yue-Ying Liu, Gemma Melisi, M. Ashwin Reddy

**Affiliations:** 1 Judith Kingston Retinoblastoma Unit, Royal London Hospital, Barts Health NHS Trust, London, UK; 2 Department of Ophthalmology, Queensland Children’s Hospital, South Brisbane, QLD Australia; 3 Queen Mary University of London, London, UK; 4 Department of Paediatrics, Moorfields Eye Hospital NHS Foundation Trust, London, UK

**Keywords:** Quality of life, Retinal diseases

Retinoblastoma presents significant psychosocial challenges alongside its life-threatening diagnosis [1, 2]. Often occurring in early childhood, treatment involves repeated examinations under anaesthesia, dilating eye drops and potential chemotherapy or enucleation [3]. These interventions can be profoundly traumatic and impact sensitive periods of cognitive and emotional development [1, 2]. While clinical efforts prioritise tumour control and vision preservation, it is essential to also address the psychological well-being of these patients [3].

Integrating purposeful play therapy into retinoblastoma care is a developmentally appropriate approach to mitigate trauma and foster emotional resilience in paediatric ocular oncology. Play therapy provides an evidence-based modality for children to externalise conflict, express emotions and process medical experiences they are not able to articulate using meaningful language [4]. This is particularly important in children adjusting to life-altering situations such as monocular vision or prosthetic eye use.

**Fig. 1:**
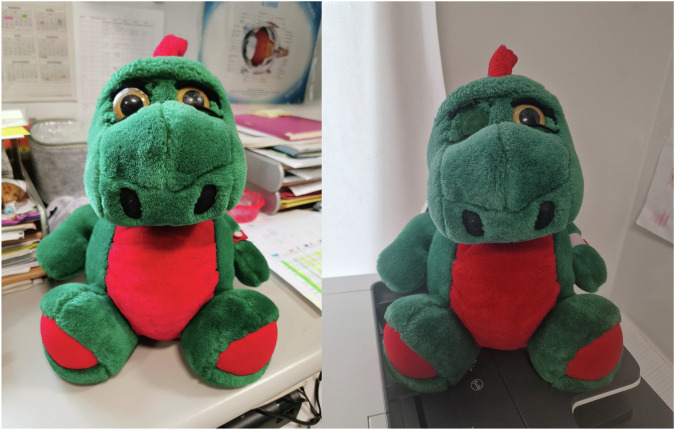
Plush toys utilised during preparation play.

At the Royal London Hospital, health play specialists provide a patient-centred approach in collaboration with a multidisciplinary team including ophthalmologists, clinical nurse specialists, psychologists and orthoptists. Play-based interventions are delivered pre- and post-procedure. Preparation play utilises plush toys with removable eyes or intravenous lines to introduce treatment in a non-threatening way, reducing anticipatory anxiety and improving cooperation (Fig. 1). Post-procedural play aims to facilitate emotional processing and reinforces coping strategies following challenging events.

Beyond direct procedural support, care is extended through innovative initiatives designed for ongoing psychosocial adaptation. The ‘Eye Club’, specifically designed for children with prosthetic eyes, provides a structured environment where peers learn and teach each other about their unique situation. Participants complete tasks to earn badges, fostering a sense of mastery and community, while families connect during shared activities. Furthermore, health play specialists help normalise retinoblastoma for classmates during school visits by addressing questions directly and creating a more understanding and supportive environment, particularly concerning prosthetic eye care.

Evidence from previous studies indicates enhanced coping in children receiving play therapy, marked by reduced procedural distress and greater adherence to treatment [4, 5]. The development of emotional and behavioural regulation fostered by play therapy can contribute to more efficient delivery of care. These benefits underscore the potential of play therapy not merely as an adjunct but as a requisite component of holistic, patient-centred retinoblastoma care.

Despite its promise, play therapy remains underutilised, often due to staffing limitations or lack of institutional recognition [5]. Addressing these barriers will aid broader implementation [5]. Further dedicated qualitative and quantitative research is warranted to formally establish these benefits and optimise integration strategies [4, 5]. As retinoblastoma survival rates increase, the quality of survivorship becomes equally important. We advocate for dedicated funding, interdisciplinary advocacy and formal integration of play therapy into treatment protocols to establish this model as a standard supportive care practice, honouring the comprehensive well-being of every child.
